# The influence of government-issued lockdowns during the COVID-19 pandemic on traumatic brain injuries in Tyrol, Austria

**DOI:** 10.1016/j.bas.2024.104159

**Published:** 2024-12-09

**Authors:** Victoria Schön, Alina Farbmacher, Lukas Grassner, Barbara Klein, Claudius Thomé, Daniel Pinggera

**Affiliations:** a Department of Neurosurgery, Medical University Innsbruck, Innsbruck, Austria; b Department of Neurosurgery, Christian Doppler Clinic, Paracelsus Medical University, Salzburg, Austria; c Institute of Molecular Regenerative Medicine, Spinal Cord Injury and Tissue Regeneration Center Salzburg, Paracelsus Medical University, Salzburg, Austria

**Keywords:** COVID-19, Traumatic brain injury, Curfews, Governmental restrictiosn

## Abstract

**Introduction:**

In February 2020, COVID-19 infections started to spread in Austria. This was followed by governmental actions and constraints such as lockdowns, quarantine protocols, and a ban on outdoor sports. The goal of this study was to investigate the influence of these measures on the number of traumatic brain injuries (TBI) in the state of Tyrol.

**Methods:**

The incidence of TBI during lockdowns and restrictions of outdoor activities in 2020 and 2021 were compared with corresponding periods in previous years. The data was retrospectively collected and analyzed.

**Results:**

During the first lockdown in the winter of 2020/2021, there was a notably lower incidence of moderate and severe TBIs compared to the corresponding period in 2019/2020 (p = 0.016). Similarly, there was a reduction in TBIs from sports accidents during this period (p = 0.010). However, when comparing other lockdown periods to the previous years, no differences were observed.

**Conclusion:**

The various governmental measures restricting mobility aimed to contain the COVID-19 pandemic but showed little influence on the number of TBI cases. Only a lockdown in the accident-prone winter months has influenced the incidence of TBIs.

## Highlights

• Exit restrictions and curfews minimally impact traumatic brain injury (TBI) cases.
• Consistent trauma cases highlight the need for a prepared healthcare system.
• Curfews minimally reduced sports accidents in winter.
• Strategic resource allocation is crucial for stable TBI care during pandemics.

## 1 Background

Worldwide, traumatic brain injury (TBI) and spinal cord injury (SCI) are the leading causes of trauma-related mortality and physical and mental disability, posing a major challenge to health care systems (Maas et al., 2022) (Rubiano et al., 2015)

While TBI remains a global problem, patient characteristics show regional differences and variations in etiology, demographics and therapeutic management. (Pinggera et al., 2021; Hukkelhoven et al., 2002). For example, in Tyrol (Austria), TBI is particularly associated with alpine sports. In addition, there has been a steady increase in the incidence of TBI in the elderly population in Tyrol, mainly due to falls from low heights in the home. (Mauritz et al., 2014).

With regard to the COVID-19 pandemic, the first cases of pneumonia caused by SARS-CoV-2 (severe acute respiratory syndrome coronavirus type 2) originated in Wuhan, China, and can be traced back to December 2009 (Shi et al., 2020). Due to the escalating number of infections in China, the situation was declared an epidemic in January 2020. Subsequently, the SARS-CoV-2 virus spread globally, leading to the emergence of the newly named COVID-19 disease (coronavirus disease 2019) (COVID-19-Pandemie; WHO, 2023a, WHO, 2023b). In February 2020, the first cases were identified in Austria, and the emergence and spread of the infection was significantly influenced by winter tourism in ski resorts. In response to the significant increase in COVID-19 cases, the Austrian government enacted the COVID-19 Measures Act on March 15, 2020. This legislation introduced restrictive measures, population lockdowns and quarantine measures (Moshammer et al., 2022a; Moshammer et al., 2022b; Bundesgestzblatt für die Republik Österreich, 2023).

**Fig. 1:**
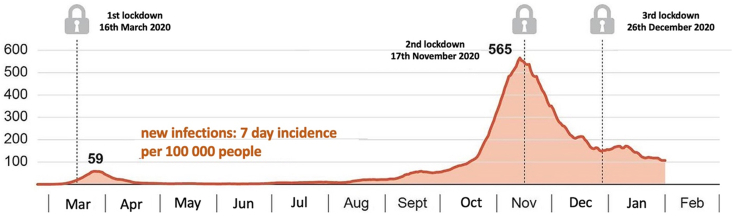
Number of COVID-19 (7-day incidence) cases in relation to the lockdowns in Austria (adapted from: Austria Presse Agentur, APA-Grafik)

In September 2020, there was a resurgence of COVID-19 infections, which led to another lockdown being imposed in November 2020. Some restrictions were relaxed around Christmas that year. This was followed by a third lockdown on December 26, 2020, which lasted until February 2021. The final lockdown began on November 22, 2021 and ended on December 11, 2021 (Hauptausschuss genehmigt bundesweiten Lockdown für, 2023; Blog 100, 2023) (see Fig. 1)

The University Hospital of Innsbruck and healthcare systems worldwide were affected by the new disease and the ensuing pandemic. New strategies had to be developed to maintain optimal patient care and minimize the risk of transmission (Pinggera et al., 2021; Nabil et al., 2022; Noureldine et al., 2020).

The primary objective of this study was to determine whether governmental quarantines had any impact on the incidence of TBI in Tyrol, Austria. This study is a continuation of a previous study by our group (Pinggera et al., 2021). In the previous study, only the first lockdown was analyzed in terms of TBI cases, and a decrease in TBI cases in Tyrol compared to pre-pandemic years was demonstrated. This led our scientific group to question whether this change in numbers could be seen in all subsequent lockdowns during the COVID-19 pandemic.

## 2 Methods

For this retrospective, single-center study, the hospital information system (KIS, Cerner Millennium®) was searched for patient records of TBI cases according to the specific inclusion criteria.

A comparison was made between the four lockdowns implemented during the COVID-19 pandemic and the corresponding calendar weeks from 2018 to 2021.

• The first lockdown in weeks 12–14 in 2020 was compared with the corresponding weeks in 2019 and 2021.
• 2nd and 3rd lockdowns, weeks 47–49 in 2020 and 2021, compared with the corresponding weeks in 2019.
• 4th lockdown, weeks 52 - 5 in 2020/2021 (over the turn of the year) was compared with the corresponding weeks in 2018/2019 and 2019/2020.

## 3 Patients

All patients treated for TBI at the Department of Neurosurgery at the University Hospital Innsbruck were included. In addition, consultations from peripheral hospitals within the state of Tyrol and referrals from other primary hospitals were included. Patients with significantly incomplete data were excluded from the study.

Medical history (cause of TBI, initial Glasgow Coma Scale (GCS) and GCS on arrival at hospital, alcohol intoxication, pupillary reaction, intracranial lesions, TBI classification, treatment, anticoagulation therapy, mortality and COVID-19 test result, and further demographic data) was collected from the medical records. Imaging data were analyzed using the first computed tomography (CT) scans after the trauma.

With regard to the cause of the accident and the geographical location in Tyrol, a distinction was made between sports injuries in general and alpine sports injuries. Alpine sports accidents included skiing and snowboarding accidents, mountain bike falls, avalanche accidents, and mountaineering and climbing accidents. Non-alpine sports injuries included road cycling and, for example, football accidents.

Lesions found on initial CT scans included epidural haematoma (EDH), acute subdural haematoma (aSDH), chronic and subacute subdural haematoma (cSDH) (the causative fall occurred during the observation period), intracranial haemorrhage (ICH), contusion, intraventricular haemorrhage (IVH), diffuse axonal injury (DAI), injuries to the skull, skull base, and skull and facial fractures, and combinations of all of these. Treatment decisions were based on the Brain Trauma Foundation (BTF) guidelines. (Carney et al.; Bullock et al., 2006).

## 4 Statistical analysis

Statistical analysis of the collected data was performed using Microsoft ® Excel ® for Microsoft 365 MSO (version 2303 Build 16.0.16227.20202), IBM SPSS Statistics [version 29 (IBM Corporation, Armonk, New York, USA)] and GraphPad Prism (version 8 for Windows, GraphPad Software, Boston, Massachusetts, USA). Pearson's chi-squared four-way test and Fisher's exact test were used to check the distribution and frequency of categorical variables over the study period. All results with a p-value <0.05 were considered statistically significant. The Shapiro-Wilk test was used to assess the normal distribution of continuous data. To compare the number of weekly TBI cases over time between 2019 and 2020, a Wilcoxon matched-pairs signed rank test was used. For normally distributed data, a 1-way ANOVA followed by a Dunnett post-hoc test was used to compare all other years to 2020, while a Kruskal-Wallis test with a Dunn multiple comparison test was used for non-parametric data.

## 5 Results

A total of 1069 patients (males: n = 713, 67%) were included during the observation period. Twenty-five patients were excluded because of missing data. The mean age at the time of TBI was 63 years (σ = 24 years, IqR: 33 years, range: 0–101).

The severity of TBI could be determined in 888 patients (mild, moderate, severe). The majority of patients had a mild TBI (n = 792, 74%). Moderate TBI was diagnosed in 72 cases (7%) and severe TBI in 205 cases (19%). The years 2019 and 2020 showed no significant difference in the distribution of TBI severity (p = 0.4). The majority of patients in each period had mild TBI, while severe TBI was the second most common diagnosis. The mortality rate in our study was 15% (n = 154).

The most common cause of TBI is falls from a low height, with a total of 690 cases (65%). In 2020, the number of cases caused by a fall from a low height was 305 (66%), slightly higher than in 2019 with 269 cases (63%). Traffic accidents were a distant second with 140 cases (13%). There was no significant difference in traffic accidents between 2019 with 63 cases (15%) and 2020 with 65 cases (14%). Alpine sports accidents came in third place, with a total of 115 cases (11%). The majority of alpine sports accidents occurred in 2020 with 50 cases (11%), while the number of cases in 2019 was 38 (9%). In a total of 4% of TBI cases, the cause was an act of violence, while in a further 3% of cases, no cause could be found in the patient's file. A fall from a great height was recorded in 3% of cases, and other sports injuries and work accidents each accounted for 1% of cases. Comparing 2019 with 2020, there were no significant differences in the distribution of accident causes (p = 0.09). Mostly, patients received primary treatment at the University Hospital Innsbruck, contrary to those treated in peripheral hospitals and managed neurosurgically solely through teleradiological referral (p < 0.001). In 2019, this amounted to 327 cases (77%) receiving treatment in Innsbruck, while peripheral hospitals managed only 96 cases (23%). A comparable distribution persisted in 2020, where 358 cases (77%) were treated in Innsbruck, and 102 cases (23%) were handled by peripheral hospitals. This trend was similarly observed in the shorter periods of 2018 and 2021.

When looking at the different pathologies, it is important to note that some individuals had combinations of several lesion patterns and therefore had multiple diagnoses. aSDH was the most commonly diagnosed lesion pattern (n = 409, 38%), followed by traumatic subarachnoid haemorrhage (tSAB) (n = 339, 32%) and skull/skull base fractures (n = 309, 29%). 213 patients (20%) had a contusion, followed by 183 individuals (17%) with a cSDH. Midface fractures were seen in 150 people (14%) and diffuse axonal damage in a further 98 (9%). ICH was diagnosed in 84 patients (8%) and EDH in 61 (6%). IVH was the least common finding (n = 2%). Comparing 2019 to 2020, all diagnoses except midface fractures were slightly more common in 2020.

When considering the treatment chosen, it should be noted that some individuals received a combination of treatments. Table 1 shows the treatment options chosen. No surgery was performed in 775 patients (72%). Comparing 2019 to 2020, the numbers remained fairly constant.

**Table 1:** Treatment options for TBI cases during the years 2018 - 2021

year	burr hole %	craniotomy %	decompressive craniotomy %	ICP monitoring %	no surgery %
**2018**	8	8	15	8	69
**2019**	12	5	4	10	74
**2020**	13	6	3	10	73
**2021**	15	9	4	11	68

**total**	13	6	4	10	72

The use of an antiplatelet drug, mainly aspirin or clopidogrel, was recorded in 194 patients (19%). The use of an oral anticoagulant was noted in 175 people (17%). A combination of both drug categories was observed in only 10 people (1%). When comparing data from 2019 to 2020, the results were largely consistent.

A total of 94 patients (9%) exhibited an elevated alcohol level during the accident, determined through either a breath alcohol test and/or blood alcohol level.

Pupil reactivity data were available for 948 patients (89%). In the majority of cases (n = 828, 87%), both pupils were reactive to light. In 53 patients (6%), only one pupil was reactive to light, while 67 patients (7%) had no pupil reactivity to light.

## 6 Lockdown-related results

**Fig. 2:**
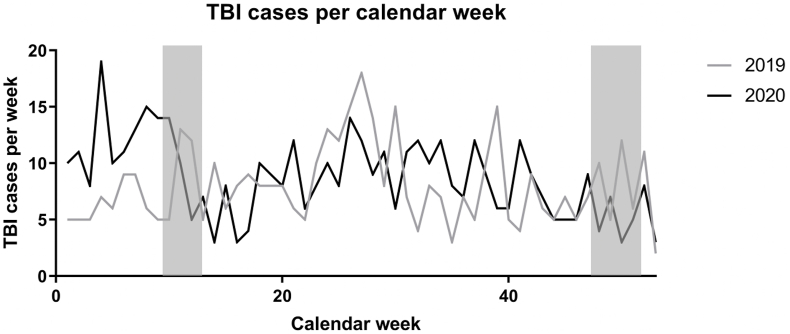
TBI cases per calendar week in 2019 und 2020 (periods of lockdown marked grey).

The median number of total TBI cases per calendar week did not differ significantly between 2019 and 2020 (p = 0.20; 2019: 7(5) and 2020: 9(5), median (IQR)). Fig. 2 shows the distribution of the total number of TBI cases per calendar week for 2019 and 2020. The blue bars indicate the period of the first two closures.

When the total number of TBI cases per calendar week was analyzed over time, there were no significant differences between 2019 and 2020 (p = 0.32). In addition, TBI cases were divided by severity, which also showed no significant difference between 2019 and 2020 in the occurrence of mild (p = 0.26) or moderate/severe (p = 0.61) TBI per calendar week over time (data not shown).

Next, the individual calendar weeks in 2020 and 2021 during which government-declared lockdowns and other government-imposed restrictions on activities occurred during the COVID-19 pandemic were analyzed. These periods were compared with the same calendar weeks in the pre-pandemic years of 2019 and 2018.

## 7 1st Lockdown

**Fig. 3:**
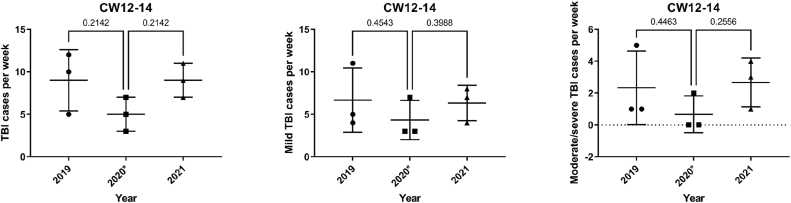
TBI cases durint the 1st curfey in the calendar weeks 12-14 in 2020 compared to the years 2019 und 2021 (∗marsk the year with the lockdown)

There were fewer TBI cases in calendar weeks 12–14 in 2020, the first lockdown in Austria, than in 2019 and 2021, but this was not statistically significant (p = 0.21) for TBI cases overall. When only mild TBI and moderate/severe TBI cases were analyzed separately, no significant differences were found (mild TBI: 2019 p = 0.45; 2021 p = 0.39; moderate/severe TBI: 2019 p = 0.45; 2021 p = 0.26) (see Fig. 3).

## 8 2nd and 3rd Lockdown

**Fig. 4:**
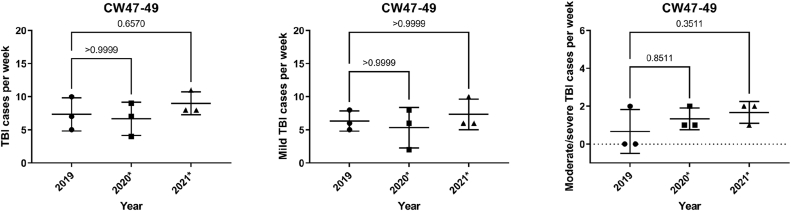
TBI cases during the 2nd and 3rd lockdown in the calendar weeks 47-49 in 2020 and 2021 compared to the years. (∗ marks the year with the lockdown)

Similar results to the 1st lockdown were shown when considering the 2nd lockdown during calendar weeks 47–49 in 2020 and the 3rd lockdown during calendar weeks 47–49 in 2021, compared to 2019. There was no statistically significant difference when considering the total number of TBI cases during the 2nd lockdown compared to the years 2019 (p > 0.99) and the 3rd lockdown in 2021 (p = 0.66). No significant difference was also found when analyzing TBI severity (mild TBI: p > 0.9 comparing 2020 with 2019 and 2021 with 2019, moderate/severe TBI: p = 0.8 comparing 2020 with 2019, p = 0.3 comparing 2021 with 2019) (Fig. 4).

## 9 4th Lockdown

**Fig. 5:**
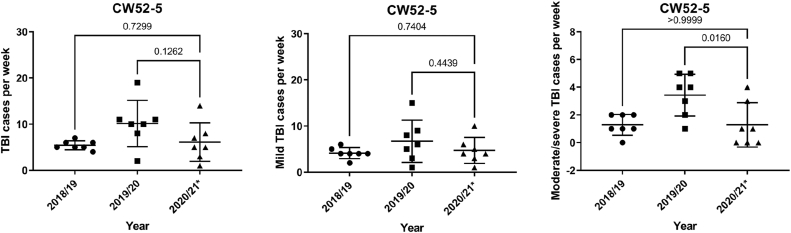
TBI cases during the 4th lockwond in the calendar weeks 52 - 05 in 2019/2020 comapred to the years 2018/19 and 2021/21 (∗ marks the year the lockdown)

When comparing the total number of TBIs during the 4th closure, which took place at the turn of the year 2020/21, a slight trend was observed when comparing 2019/20 and 2020/21 (p = 0.13), but no significance could be reached for 2018/19 compared to 2019/20 (p = 0.73). However, there was a statistically significant reduction in the number of moderate and severe TBIs (p = 0.02) (Fig. 5).

**Fig. 6:**
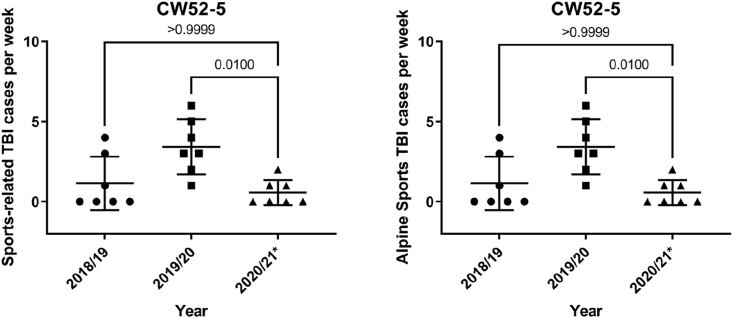
Sport releated TBI cases during the 4th lockdown in the calendar ceeks 52- 05 in 2019/20 compared to the years 2018/19 and 2020/2021.(∗ marks the year with the lockdown)

There was also a significant difference in sports and alpine sports accidents. In the fourth quarantine in 2020/21, these cases were significantly lower than in the same period in 2019/20 (p = 0.01) (Fig. 6).

Table 2 shows a comparison between TBI causes and TBI severity for each period of the lockdowns. During the first two bans, the number of TBI cases is lower than in the other years observed. Furthermore, no cases of TBI related to alpine sports were found during the first two bans. The number of alpine sports-associated accidents increased slightly in the comparison periods of 2019 and 2021.

**Table 2:** TBI causes and severity during the different lockdwons during the COVID-19 pandemic. (grey marks the year with the pandemic)

year	alpine sport accidents (n)	other sport accidents (n)	low impact falls (n)	traffic accidents (n)	mild TBI (n)	moderate TBI (n)	severe TBI (n)
1st curfew (calendar weeks 12–14)							
**2020**	0	1	14	0	13	0	2
**2019**	5	1	15	1	22	1	5
**2021**	5	0	16	2	19	1	7

**2**nd **and 3**rd **curfew (calendar weeks 47**–**49)**							
**2020**	0	0	14	2	14	1	2
**2019**	2	0	14	1	20	1	1
**2021**	2	0	17	1	21	0	3

**4th curfew (calendar weeks 52**–**05)**							
**2020/21**	6	0	30	1	34	4	5
**2018/19**	10	0	24	4	33	3	6
**2019/20**	26	0	43	1	50	7	17

## 10 Discussion

The aim of this study was to investigate whether the COVID-19 pandemic, with its associated lockdowns and government restrictions, had an impact on the incidence of TBI in a specific region (Tyrol, Austria). Given the stringent government measures, including lockdowns, quarantine and a ban on outdoor sports, one might have expected a significant change in the number of TBI cases. Specifically, a decrease in TBI cases was expected in 2020, coinciding with the onset of the COVID-19 pandemic and the first implementation of governmental lockdowns, compared to previous years. In addition, a shift in the causes of TBI was expected, such as a decrease in outdoor sports and traffic-related incidents and an increase in household accidents. However, we observed minimal impact of the above government measures on the incidence of TBI cases.

Of course, these are only TBI cases in which the neurosurgical department in Innsbruck was involved. Neurosurgery is not involved in mild TBI cases, which remain in the peripheral hospitals or are treated by the in-house trauma department. This is therefore only an analysis of TBIs treated by neurosurgery. If the data of all mild TBIs could be collected and included in the data analysis, the results might be different.

The expectation of an overall decrease in TBI cases during the pandemic was not confirmed by this data analysis. There were no significant differences in the number of cases or in the severity of TBI. Only during the third lockdown in calendar weeks 52 to 05 in 2020/2 was a slight reduction in moderate and severe TBI cases per week observed, compared with the same calendar weeks in 2019/20. In addition, there was a significant reduction in sports- and alpine-related TBI cases during the same period. In the other closed areas, the number of TBI cases was lower, but not statistically significant. It is worth noting that in 2019 and 2021, the number of alpine sports accidents increased slightly in the comparison periods. One reason could be that the peak season of many Tyrolean ski resorts is between December and March, and government measures were introduced during the peak season. (Steiger and Scott) Therefore, the third hard closure seems to be the most suitable to observe changes in alpine accidents. It should also be mentioned that during the first and second lockdowns, ski resorts were closed. During the third lockdown, however, the ski resorts were allowed to reopen to the local population. This seems to have influenced the number of documented winter sports accidents (Blog 79, 2023; Blog 112, 2023).

Throughout the pandemic, the impact of COVID-19 infection on trauma cases has been documented and studied worldwide. Findings varied due to regional differences in infrastructure, available resources and different political and geographical conditions. In addition, governmental COVID-19 policies varied widely; in Austria alone, each province implemented its own set of policies (Blog 112, 2023; Blog 157, 2023).

A review of Austrian accident data from 2020 during the COVID-19 pandemic showed a reduction in traffic volume of more than 60% during the initial closure. Although the number of traffic accidents decreased during this period, a comparison of the number of kilometres travelled with the number of fatal accidents showed an increase compared to 2019 (Straßenverkehrsunfälle mit Personenschaden, 2020, 2020). Over 50% of incidents during the initial lockdown occurred in residential areas, with a notable increase in fall-related injuries, particularly among those aged 65 and over. In addition, an increase in alcohol-related accidents was observed (Der Corona-Effekt - Auswirkungen der Covid-19-Pandemie auf die Lebensbereiche Verkehr & Mobilität and Sport & Haushalt sowie Eigentumsschutz).

International studies have shown variations in the incidence of traumatic brain injury (TBI) during the COVID-19 pandemic, with some reporting an increase, others a decrease, and still others no change.

Two Finnish groups found no change in TBI cases during the lockdowns. One group compared the number of patients in the neurointensive care unit at Helsinki University Hospital and found a constant cohort of patients compared with previous years. The study concluded that the pandemic lockdowns did not affect the number of TBI cases (Luostarinen et al., 2020). The second analyzed data from four Finnish hospitals on the incidence and characteristics of severe TBI during the first lockdown. In this study, the incidence of severe TBI remained the same compared to the years 2016–2018 (Riuttanen et al., 2021). These findings were supported by a study from Rwanda (Tang et al., 2023).

In contrast to the stable patient numbers observed in other studies, there was an increase in TBI cases related to motor vehicle accidents and falls in the Czech Republic. However, a decrease was observed in skiing-related accidents (Berková et al., 2022). Furthermore, a meta-analysis of 13 international studies found an increased TBI mortality rate in low- and middle-income countries compared with the pre-pandemic period. This increase is likely due to a higher number of COVID-19 infections and limited medical care in developing countries (Damara et al., 2022). These findings have been confirmed by colleagues in Indonesia (Prawiroharjo et al., 2020).

In a previous study, our group demonstrated a decrease in TBI cases during the initial quarantine period in Tyrol compared to pre-pandemic years (Pinggera et al., 2021). This discrepancy with the current study may be due to the comparison of different pre-pandemic periods with the "during COVID" data. Other European studies, including those conducted in Italy and the Netherlands, also reported a decrease during the initial lockdown period (Munari et al., 2021; Santing et al., 2020). In Berlin, a comparison of neurological emergencies between February and April in 2019 and 2020 showed an overall decrease in neurological emergencies, although traumatological cases remained constant (Hecht et al., 2020). In particular, India documented a decrease in motor vehicle accidents, falls, and mild to moderate traumatic brain injury (TBI). However, there was an increase in assaults and severe TBI cases during the pandemic (Rajalu et al., 2022). Similar findings were observed in Pennsylvania during the first lockdown compared with 2017–2019, with a shift in accident mechanisms, including more home injuries, particularly falls and gunshot wounds. However, the distribution of TBI severity remained unchanged (Algattas et al., 2021).

All of the above studies compared TBI figures with the corresponding periods before the pandemic. This study analyzed TBI cases during the pandemic lockdowns. It is possible that the number of TBI cases themselves did not change much because many people were unable to work and had to stay at home. Therefore, people spent more time indoors, which could have affected the number of TBI cases caused by home accidents.

As this is a retrospective study, there are some limitations. It is conceivable that some TBI cases may have gone unrecorded, particularly mild cases, as people may have avoided hospital visits during the pandemic due to concerns about COVID-19 infection (Lester et al., 2021). However, as the University Hospital of Innsbruck is the only level 1 trauma centre for neurotrauma in the state of Tyrol, it is highly likely that all moderate and severe TBI cases were recorded.

Another limiting factor is the variability in the implementation of government policies during lockdowns. In addition, external factors such as weather conditions, snowfall and slope conditions were not taken into account for their potential impact on winter sports accidents.

Taken together, these studies and our own research suggest that better training of medical professionals, enforcement of safety protocols and strategic allocation of resources could improve the quality of patient care in future international health crises.

## 11 Conclusion

Government-imposed restrictions and lockdowns appear to have little effect on the incidence of TBI. Only a slight decrease was observed during the accident-prone winter months.

The consistent number of trauma cases during periods marked by various lockdowns underscores the need for the health system to remain equipped to effectively manage such events during pandemics or other public health crises. Strategic resource allocation, continued refinement of safety protocols and guidelines, streamlined surgical management and comprehensive staff training are essential to ensure optimal care for all patients in potential future public health emergencies.

## Declaration of competing interest

The authors declare that they have no known competing financial interests or personal relationships that could have appeared to influence the work reported in this paper.
